# Glioblastomes intra médullaire chez l'enfant: localisation et type histologique rare

**DOI:** 10.11604/pamj.2017.26.91.8171

**Published:** 2017-02-23

**Authors:** Abderrazzak El Saqui, Aggouri Mohamed

**Affiliations:** 1 Service de Neurochirurgie, CHU Hassan II, Fès, Maroc

**Keywords:** Glioblastomes, moelle épinière, intra médullaire, rachis dorsal, Angioma, aggressive, spine, MRI, urgery

## Image en médecine

Un enfant de 4 ans, sans antécédents, qui a présentée deux mois avant son admission, des céphalées associées à des douleurs cervicales irradiant vers les épaules et le rachis dorsal, aggravées par l'installation d'une lourdeur des quatres membres, chez qui l'examen a trouvé un enfant conscient, avec une tétraparésie lourde. L'IRM médullaire a objectivée un processus tumoral médullaire cervicale étendu de la jonction bulbo-médullaire jusqu'à D1 en hyposignal T1 et en hypersignal T2 avec une composante charnue et kystique rehaussée de façon hétérogène après injection de gadolinium (A). La patiente a bénéficié d'une laminectomie de 8 niveau, de C1 à D1, avec craniectomie à os perdu sous occipitale et exérèse macroscopiquement totale du processus. Un examen extemporané est revenu en faveur de glioblastome, diagnostic qui a été confirmé par l'examen anatomo-pathologique de la pièce opératoire (B). La patiente a bénéficiée de la radiothérapie post opératoire. L'évolution a été marquée par l'amélioration progressive de la tétraparésie avant son décès par des troubles neuro-végétatifs 18 mois plus tard. Les glioblastomes de la moelle épinière sont exceptionnelle, L'IRM après injection du Gadolinium reste la meilleure modalité d'imagerie pour le diagnostic (C). Le traitement de choix est l'exérèse chirurgicale la plus complète possible suivie d'une radiothérapie et éventuel chimiothérapie, La survie moyenne de ces patients est de 12 mois après le diagnostic.

**Figure 1:**
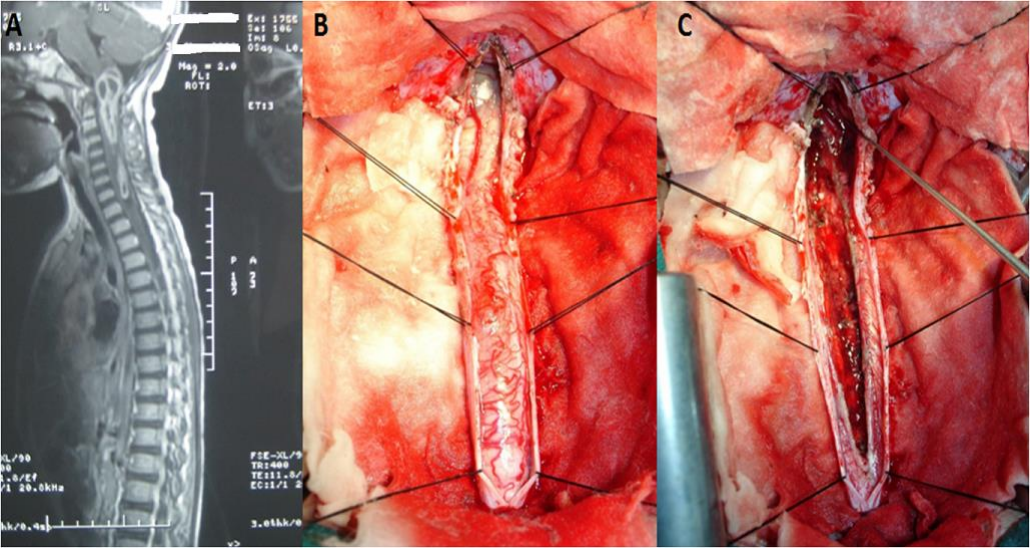
A) IRM cervicale en coupe sagitale T1 objectivant une lésion siégeant au niveau de la jonction bulbo-medullaire étendue jusqu’à la première vertèbre dorsale prenant le contraste en périphérie après injection de Gadolinium; B) aspect per-opératoire de la jonction bulbo-médullaire après réalisation d’une laminectomie des vertèbres cervicales et craniectomie occipitale avec ouverture et suspension de la dure-mère montrant la moelle cervicale avec un processus grisâtre au niveau de la jonction bulbo-médullaire; C) aspect per-opératoire de la jonction bulbo-médullaire après myélotomie postérieure avec exérèse totale du processus tumoral

